# The Effect of Exercise on the Early Stages of Mesenchymal Stromal Cell-Induced Cartilage Repair in a Rat Osteochondral Defect Model

**DOI:** 10.1371/journal.pone.0151580

**Published:** 2016-03-11

**Authors:** Shoki Yamaguchi, Tomoki Aoyama, Akira Ito, Momoko Nagai, Hirotaka Iijima, Junichi Tajino, Xiangkai Zhang, Wataru Kiyan, Hiroshi Kuroki

**Affiliations:** 1 Department of Motor Function Analysis, Human Health Sciences, Graduate School of Medicine, Kyoto University, Kyoto, Japan; 2 Japan Society for the Promotion of Science, Tokyo, Japan; 3 Department of Development and Rehabilitation of Motor Function, Human Health Sciences, Graduate School of Medicine, Kyoto University, Kyoto, Japan; 4 Department of Orthopaedic Surgery, Graduate School of Medicine, Kyoto University, Kyoto, Japan; 5 Congenital Anomaly Research Center, Graduate School of Medicine, Kyoto University, Kyoto, Japan; University of Rochester, UNITED STATES

## Abstract

The repair of articular cartilage is challenging owing to the restriction in the ability of articular cartilage to repair itself. Therefore, cell supplementation therapy is possible cartilage repair method. However, few studies have verified the efficacy and safety of cell supplementation therapy. The current study assessed the effect of exercise on early the phase of cartilage repair following cell supplementation utilizing mesenchymal stromal cell (MSC) intra-articular injection. An osteochondral defect was created on the femoral grooves bilaterally of Wistar rats. Mesenchymal stromal cells that were obtained from male Wistar rats were cultured in monolayer. After 4 weeks, MSCs were injected into the right knee joint and the rats were randomized into an exercise or no-exercise intervention group. The femurs were divided as follows: C group (no exercise without MSC injection); E group (exercise without MSC injection); M group (no exercise with MSC injection); and ME group (exercise with MSC injection). At 2, 4, and 8 weeks after the injection, the femurs were sectioned and histologically graded using the Wakitani cartilage repair scoring system. At 2 weeks after the injection, the total histological scores of the M and ME groups improved significantly compared with those of the C group. Four weeks after the injection, the scores of both the M and ME groups improved significantly. Additionally, the scores in the ME group showed a significant improvement compared to those in the M group. The improvement in the scores of the E, M, and ME groups at 8 weeks were not significantly different. The findings indicate that exercise may enhance cartilage repair after an MSC intra-articular injection. This study highlights the importance of exercise following cell transplantation therapy.

## Introduction

Articular cartilage repair is one of the most challenging issues in the orthopedic field, owing to the poor intrinsic healing capacity of articular cartilage.[1] In the clinical setting, debridement,[2] microfracture,[3] and autologous tissue transplantation therapy [4] have been used to treat cartilage defects. However, some problems, such as the lack of compatibility with host tissue, exist.[5] The cartilage regenerated by most of those methods is similar to fibrocartilage.[6–8] The recently introduced cell supplementation therapy is one of the methods expected to treat cartilage defects. One of the candidate cells for supplementation is autologous chondrocytes [8, 9] and multipotent stem cells such as mesenchymal stem or stromal cells. Mesenchymal stromal cells (MSCs) also represent an attractive cell source for cartilage repair based on their multipotency as well their ability to undergo chondrogenic differentiation, they secrete some kind of cytokine, they are easy isolate, and they have an immune tolerance.[10, 11] Although some studies reported the cell types and supplement methods used, such as intra-articular injection,[12] biomaterial scaffolds,[13] and growth factor addition,[14] only a few reports have verified the efficacy and safety of exercise and rehabilitation therapy after cell supplementation. In patients with osteoarthritis, exercise yields a beneficial effect for degenerative cartilage,[15, 16] pain, stiffness, and physical function.[17] The use of exercise as a rehabilitation procedure prevents the development of disuse syndrome, improves activities of daily living, and enables an earlier return to sports. Weight bearing in rehabilitation following matrix-associated autologous chondrocyte implantation for patients with focal full thickness cartilage defects led to good clinical outcomes as evaluated by MRI.[18] Although rehabilitation after cell supplementation has been confirmed to be important, little evidence is available on the effect of exercise or physical stimulation after cell supplementation therapy histologically. *In vitro*, physiological mechanical stress stimulates the differentiation of MSCs into chondrocytes, extracellular matrix (ECM) synthesis, cytokine secretion, [19] and cell adhesion; however, excessive stress might inhibit progenitor cell proliferation [20] and cause cell death and matrix degeneration.[21] *In vivo*, appropriate timing of treadmill exercise after a full thickness cartilage defect could promote the healing of cartilage defect. In contrast, inappropriate exercise may inhibit cartilage repair after cell supplementation.[22] We hypothesized that an appropriate level of exercise will promote the repair of osteochondral defects through cartilage formation following cell supplementation procedures such as the intra-articular injection of MSCs. In the present study, we aimed to assess the effect of exercise after the intra-articular injection of MSCs on osteochondral defects in a rat model.

## Materials and Methods

### Animals

This experiment was approved by the animal research committee of Kyoto University. Fifty-three 8-week-old male Wistar rats were used in this study. Forty-eight rats were used as an osteochondral defect model for the experiment, two were used as a baseline model for osteochondral and normal cartilage, and three were utilized as MSC donor rats. The mean body weight of the animals was 187.9 ± 6.88 g (mean ± standard deviation). They were kept in standard plastic cages in a temperature-controlled room, were fed a standard diet, and were given free access to tap water.

### Surgical procedure for the creation of an osteochondral defect

The rats were anesthetized with somnopentyl (0.1 ml/100 g, Kyoritsu Seiyaku Co., Tokyo, Japan) followed after inhalation anesthesia induced by isoflurane. A parapatellar skin incision was made on the medial side of both knee joints. The patella was dislocated laterally to provide access to the femoral groove. An osteochondral defect (1 mm in diameter and reaching subchondral bone) was created at the center of the femoral groove with the knee flexed using a 1-mm biopsy punch. After the patella was relocated, the wound was closed with interrupted 6–0 nylon sutures. After the operation, the rats were kept warm at 37°C on heat insulating board. However we didn’t induce any analgesic drug during on post-surgery periods.

### MSC isolation and injection

Euthanasia was induced by somnopentyl, and the skin over the femoral bone of the MSC donor rat was disinfected. An incision was made, and the femur was isolated. Using an 18-gauge needle, bone marrow was aspirated and mixed from three donor rats. Mixed bone marrow was suspended in a medium containing MEM alpha + GlutaMAX (Gibco, California, USA) that included 10% fetal bovine serum (Hyclone, Logan, USA), 50 U/mL penicillin (Nacalai Tesque Inc., Kyoto, Japan), and 50 μg/mL streptomycin (Nacalai Tesque). The bone marrow cells were seeded onto a 100-mm culture dish and incubated in a humidified 5% CO_2_/95% air atmosphere at 37°C for 24 h. After the first 24 h of incubation, the medium was changed every 3 days. The cells eventually proliferated to a confluence rate of 70–90%, after which they were harvested and cultured until passage 3. Subsequently, the adherent cells were rinsed with phosphate-buffered saline (PBS), treated with 0.25% trypsin, and 0.02% ethylenediaminetetraacetic acid (EDTA). All cells were rinsed with culture medium and PBS, after which they were counted using a hemocytometer. The rats in which osteochondral defects were created were allowed to roam freely in their cages for 4 weeks to ensure that the defects became a chronic condition.[23] When the rats were 12 weeks old, 1.0 × 10^6^ MSCs were suspended in 50 μL of PBS and injected into the right knee with a 26-gauge needle under anesthesia with isoflurane. In the left knee, 50 μL of PBS was injected as a control. Chondrogenic potential of the bone marrow MSCs was examined in a pilot study.

### Exercise protocol

After the intra-articular injection, the 48 rats were randomly divided into 2 groups: exercise (n = 24) and no exercise (n = 24). The exercise protocol was started 2 days after the intra-articular injection when the rats were 12 weeks old. The rats in the exercise group were subjected to exercise 5 days a week for 2, 4 or 8 weeks using a motor-driven treadmill designed for rodents (Natsume Seisakusho Co., Tokyo, Japan) at a constant speed of 12 m/min for 30 min.[24] When not exercising, the rats were allowed to move freely in standard cages. The rats in the no exercise group were allowed to move freely in standard cages 24 h a day during the 2-, 4- or 8-week duration of the study.

### Histological assessment

The knees were classified into 4 groups: no exercise with PBS as control group (C group: left knee), exercise with PBS group (E group: left knee), no exercise with MSC injection group (M group: right knee), and exercise with MSC injection group (ME group: right knee). Following euthanasia induced by somnopentyl at 2, 4 or 8 weeks after the intra-articular injection, the femur was isolated and fixed in 4% formaldehyde in PBS (pH 7.4) overnight at 4°C. The specimens were then evaluated macroscopically and decalcified in 10% EDTA (pH 7.4). The femur was embedded in paraffin wax and cut into 6-μm-thick sections in 400–600 μm area from the edge of the defect hole. The sections were stained with hematoxylin/eosin (HE) and safranin-O/fast green (SO), and histological grading of the regenerated cartilage was performed on each section according to the histologic grading scale for defective cartilage (Table 1).[25]

**Table 1:** Wakitani cartilage repair scoring system [25].

**Cell morphology**		
	Hyaline cartilage	0
	Mostly hyaline cartilage	1
	Mostly fibrocartilage	2
	Mostly non-cartilage	3
	Non-cartilage	4
**Matrix staining intensity (metachromasia)**		
	Normal (compared with host adjacent cartilage)	0
	Slightly reduced	1
	Markedly reduced	2
	No metachromatic stain	3
**Surface regularity (total smooth area compared with entire area of cartilage defect)**		
	Smooth (>3/4)	0
	Moderate (>1/2-3/4)	1
	Irregular (1/4-1/2)	2
	Severely irregular (<1/4)	3
**Thickness of cartilage (compared with that of surrounding cartilage)**		
	>2/3	0
	1/3-2/3	1
	<1/3	2
**Integration of donor with host adjacent cartilage**		
	Both edges integrated	0
	One end integrated	1
	Neither edge integrated	2

### Immunohistochemical analysis

Serial sections were stained immunohistochemically to detect type II collagen by following the methods for immunohistochemical (IHC) analysis. The deparaffinized sections were immersed in 0.3% H_2_O_2_ to block endogenous peroxidase activity. The sections were then treated with 1.25% hyaluronidase (Sigma-Aldrich Co, St Louis, Missouri, USA) in PBS for 60 min at room temperature. After rinsing with PBS, the sections were blocked with 1.5% normal goat serum for 60 min at room temperature and incubated with a mouse monoclonal antibody directed against type II collagen (diluted 1:100; fine chemical Co., Toyama, Japan) overnight at 4°C. The sections were then rinsed in PBS and treated with horse biotinylated anti-mouse IgG (diluted 1:200; Vector laboratories, Burlingame, California) for 30 min at room temperature. To detect type I collagen, the sections were immersed in 3% H_2_O_2_ to block endogenous peroxidase activity. After rinsing with PBS for 5 min, the sections were blocked with 10% normal goat serum for 60 min at room temperature and incubated with a purified rabbit antibody directed against type I collagen (diluted 1:200; Vector laboratories, Burlingame, California) overnight at 4°C. The sections were rinsed in PBS and treated with horse biotinylated anti-mouse IgG (diluted 1:200; Vector laboratories, Burlingame, California) for 30 min at room temperature. The reaction for visualization was performed using an avidin-biotin-peroxidase system (Vecstain Elite ABC kit; Vector laboratories, Burlingame, California), and the sections were colored with a freshly prepared diaminobenzidine solution. The sections that were immunohistochemically stained for type I and II collagen were observed by employing light microscopy. In order to analyze type II collagen IHC stained sections quantitatively, pictures in TIFF format were taken at 100 times magnification and were converted to grayscale using ImageJ software. These converted images were analyzed for the % area stained by type II collagen for the presence of regenerative changes in cartilage area. The repaired area traced manually in the image software ImageJ.

### Statistical analysis

The differences in the Wakitani cartilage repair score and type II collagen quantitative percentage area among the groups for each time period were determined statistically using the Kruskal–Wallis test with a subsequent post-hoc analysis using the nonparametric Steel–Dwass test. The Wakitani cartilage repair score was histologically evaluated by a single observer in a blinded manner, and the inter-rater reliability score for the evaluation was excellent (intraclass correlation coefficient: 0.97). The software program JMP11 (SAS Institute, Cary, NC USA) was used for the statistical analysis. Alpha was set at 0.05 and descriptive statistics were calculated as the median and interquartile range.

## Results

### Histological assessment

**Fig 1:**
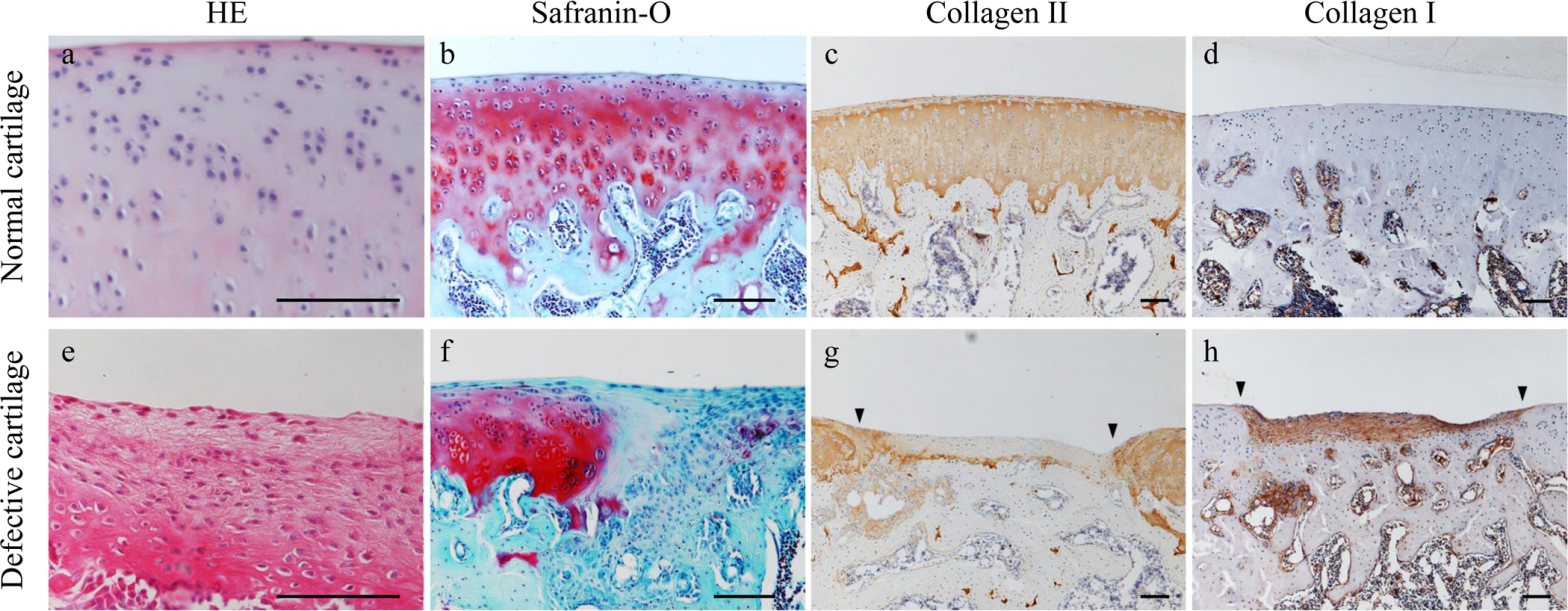
Microscopic-stained images of normal and defective cartilage. a–d: Normal cartilage. a: Stained with hematoxylin and eosin (HE; × 400); b: Stained with safranin-O/fast green (SO; ×200); c: Type II collagen immunohistochemically stained image (×100); d: Type I collagen immunohistochemically stained image (×100). Hyaline cartilage cell morphology resembling a round shaped cell was observed. Cartilage 4 weeks after the creation of an osteochondral defect. e: Stained with hematoxylin and eosin (HE; × 400); f: Stained with safranin-O/fast green (SO; ×200); g: Type II collagen immunohistochemically stained image (×100); h: Type I collagen immunohistochemically stained image (×100). Fibroblastic cell morphology resembling a spindle shaped cell was observed. Black bars represent 0.1 mm. Arrowheads denote the border between the defect area and host cartilage.

Normal cartilage and repaired cartilage 4 weeks after the creation of an osteochondral defect as a baseline were presented in Fig 1. There was defective tissue in the affected area at baseline, which was stained with type I collagen IHC but without SO stained and type II collagen IHC stained.

Macroscopic observation at each end point did not show any sign of infection or immunological rejection.

**Fig 2:**
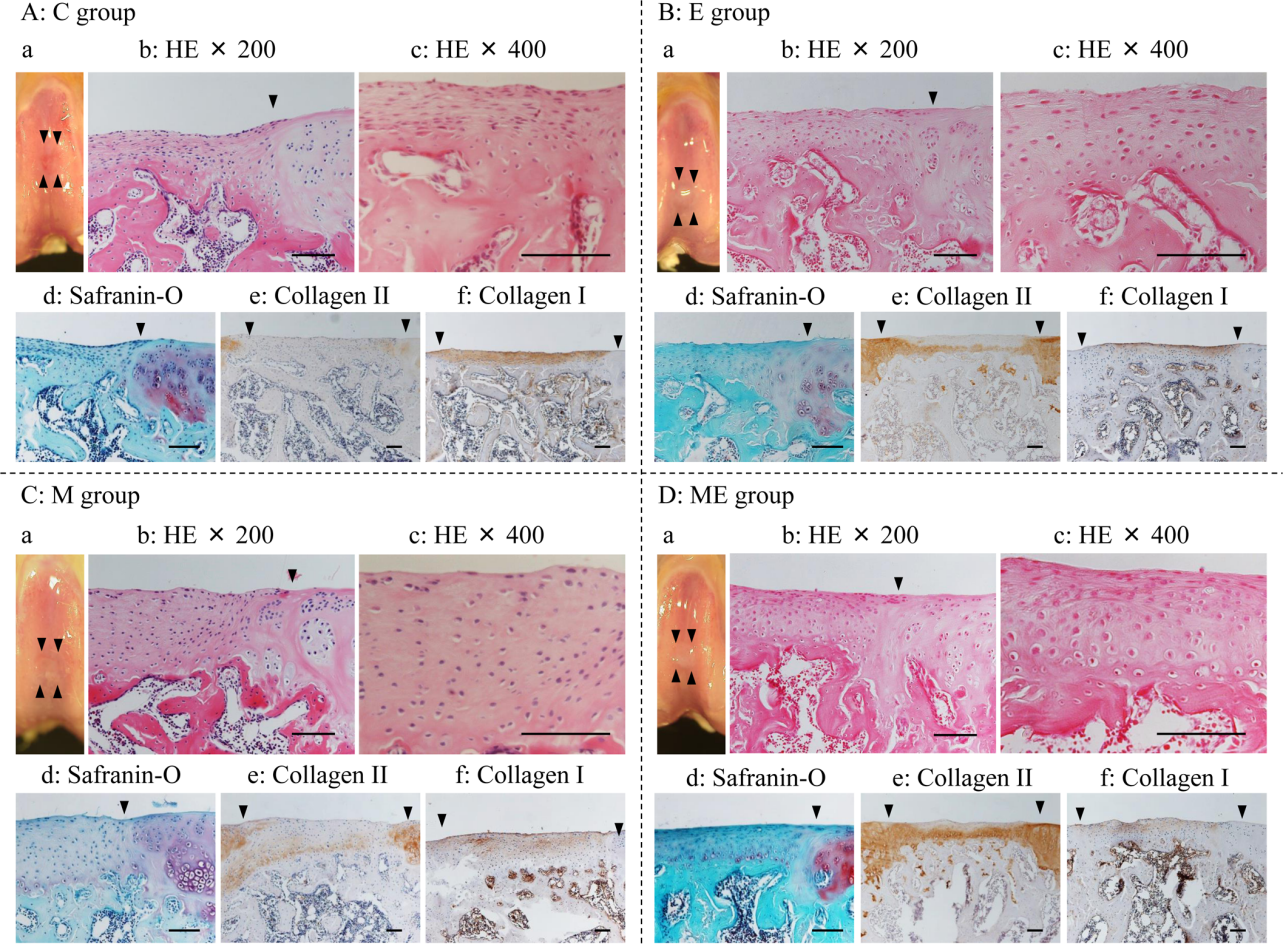
Histological images at 2 weeks. Macroscopic image and microscopic images of regenerated cartilage at 2 weeks. A: C group (Control); B: E group (exercise without MSC); C: M group (no exercise with MSC injection); D: ME group (exercise with MSC injection). a: macroscopic image. The arrow heads pointed edges of the defect; b: stained with hematoxylin and eosin (HE; ×200); c: stained with HE (×400); d: stained with safranin-O/fast green (×200); e: type II collagen immunohistochemically stained image (×100); f: type I collagen immunohistochemically stained image (×100). The arrow heads in b, d-f denote the border between the defect area and host cartilage. Black bars represent 0.1 mm.

Two weeks after MSC/PBS injection (6 weeks after the creation of an osteochondral defect), the margin of the defects in the C and E group were clearly recognized by macroscopically and the surface were slightly irregular. The repaired tissue in the C (Fig 2A) and E group (Fig 2B) was histologically composed of fibrous-like tissue that had fibroblastic cell morphology and stained negative with SO and type II collagen staining, but with IHC of type I collagen. The surface of the repaired tissue was irregular and thinner than the host adjacent cartilage. Immature bone, which was stained by type II collagen IHC, was expressed in subchondral bone on the repaired tissue in 2 samples in both groups. In the M group (Fig 2C), the margins of the defected areas were partly recognized macroscopically and the surfaces were slightly irregular. The repaired tissue displayed a fibroblastic- and hyaline-like cartilage mixed cell morphology, and the tissue exhibited markedly reduced SO staining intensity. In the IHC analysis, there was low intensity for type II collagen IHC staining in the repaired tissue in the deep zone and a wide area of expression for type I collagen IHC staining. Surface irregularity of the defects could still be noted. The channels were observed in subchondral bone on repaired tissue. In the ME group (Fig 2D), the margin of the defect area were a partly recognized by macroscopically and the surface were slightly irregular. The repaired tissues in the ME group were thicker than those in the C group, and high staining intensity was observed for IHC of type II collagen. There was only a small amount of Type I collagen IHC staining intensity observed in the ME group compared with other groups. Subchondral bone from the repaired tissue was observed.

**Fig 3:**
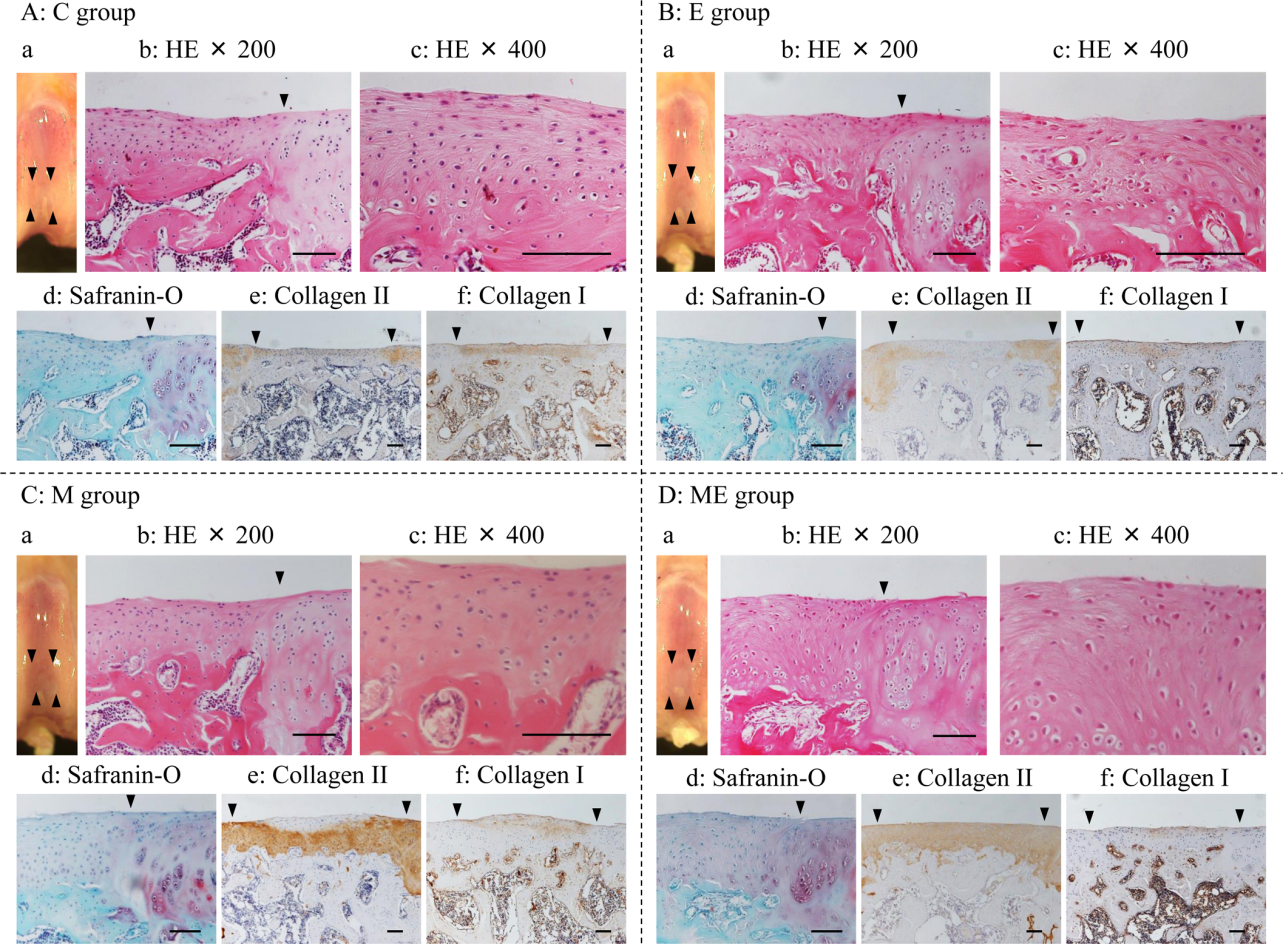
Histological images at 4 weeks. Macroscopic image and microscopic images of regenerated cartilage at 4 weeks. A: C group (Control); B: E group (exercise without MSC); C: M group (no exercise with MSC injection); D: ME group (exercise with MSC injection). a: macroscopic image. The arrow heads pointed edges of the defect; b: stained with hematoxylin and eosin (HE; ×200); c: stained with HE (×400); d: stained with safranin-O/fast green (×200); e: type II collagen immunohistochemically stained image (×100); f: type I collagen immunohistochemically stained image (×100). The arrow heads in b, d-f denote the border between the defect area and host cartilage. Black bars represent 0.1 mm.

Four weeks after MSC/PBS injection (8 weeks after the creation of an osteochondral defect), the margin of the defects in the C (Fig 3A) and E group (Fig 3B) were clearly recognized macroscopically and the surfaces were slightly irregular. The repaired tissue in the C and E groups were hardly improved histologically, such hat fibrous-like cartilage was present, and the tissue was negative for SO and type II collagen IHC staining in the area from the surface to the middle zone. The surface of the repaired tissue was still irregular and thinner than the host adjacent cartilage. Immature bone, which IHC stained for type II collagen was still expressed in the subchondral bone in the repaired tissue in C group. In the M group (Fig 3C), the margin of the defects were partly recognized macroscopically and the surface was partially smooth. With respect to the histochemical observation, fibroblastic cell morphology and tissue were reduced in the repaired tissue compared with the C group, but the tissue still exhibited markedly reduced SO staining intensity. In the IHC analysis, the repaired tissue was intensely stained for type II collagen between the middle and deep zones and type I collagen was present between the surfaces to the middle zone. In the ME group (Fig 3D), the margins of the defects were partly recognized macroscopically and the surfaces were almost smooth. With respect to the histochemical observation, the SO staining intensity increased slightly but the cell morphology indicated primarily hyaline-cartilage and the IHC staining indicated the presence of type II collagen localized in the middle in the deep zones in addition to type I collagen between the surfaces to middle zone.

**Fig 4:**
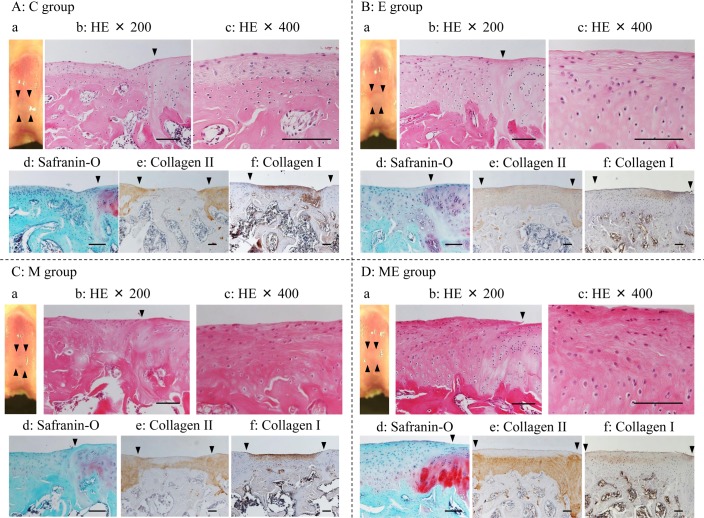
Histological images at 8 weeks. Macroscopic and microscopic images of regenerated cartilage at 8 weeks. A: C group (Control); B: E group (exercise without MSC); C: M group (no exercise with MSC injection); D: ME group (exercise with MSC injection). a: macroscopic image. The arrow heads pointed edges of the defect; b: stained with hematoxylin and eosin (HE; ×200); c: stained with HE (×400); d: stained with safranin-O/fast green (×200); e: type II collagen immunohistochemically stained image (×100); f: type I collagen immunohistochemically stained image (×100). The arrow heads in b, d-f denote the border between the defect area and host cartilage. Black bars represent 0.1 mm.

Eight weeks after MSC/PBS injection (12 weeks after the creation of an osteochondral defect), the C group (Fig 4A), did not indicate clearly repaired tissue either histologically or through and the IHC analysis. The repaired tissues were fibrocartilage like which was stained with IHC for type I collagen but without IHC of type II collagen and SO stained. Immature bone, which IHC stained for type II collagen was still expressed in the subchondral bone in the repaired tissue in C group. In the E group (Fig 4B), the margin of the defects was partly recognized macroscopically and the surfaces were partially smooth. The histochemical observation suggested that the repaired tissues were thick and the IHC suggests it was of type II collagen intensity in a wide area of the repaired tissue. In the M (Fig 4C) and ME group (Fig 4D), the margin of the defects were slightly recognized macroscopically and the surfaces were almost smooth. The cell morphology displayed hyaline-like cartilage and thick type II collagen IHC staining intensity. The IHC of Type II collagen expression on the surface region of the repaired tissue in the ME group was slightly reduced.

### The Wakitani cartilage repair score and the quantitative evaluation for area% of type II collagen IHC staining

**Fig 5:**
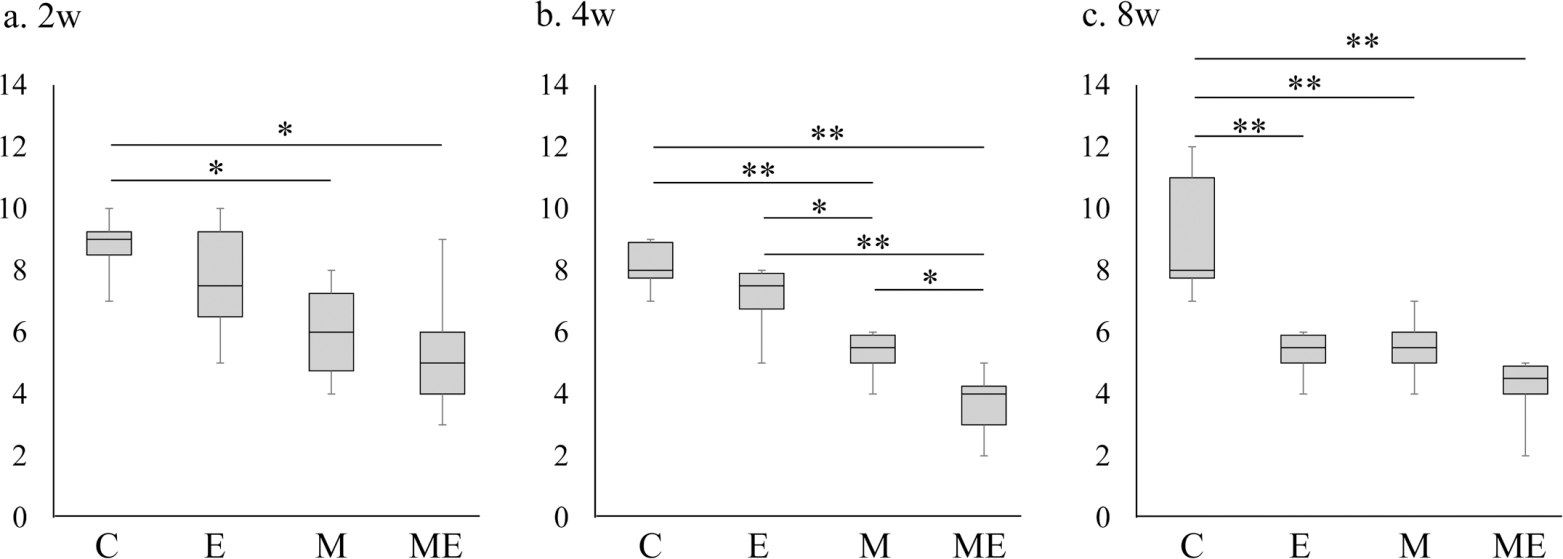
Wakitani cartilage repair score. a: At 2 weeks, b: At 4 weeks, c: At 8 weeks. Boxplots display the median and interquartile range, n = 8/group, (*P < 0.05, **P < 0.01).

**Fig 6:**
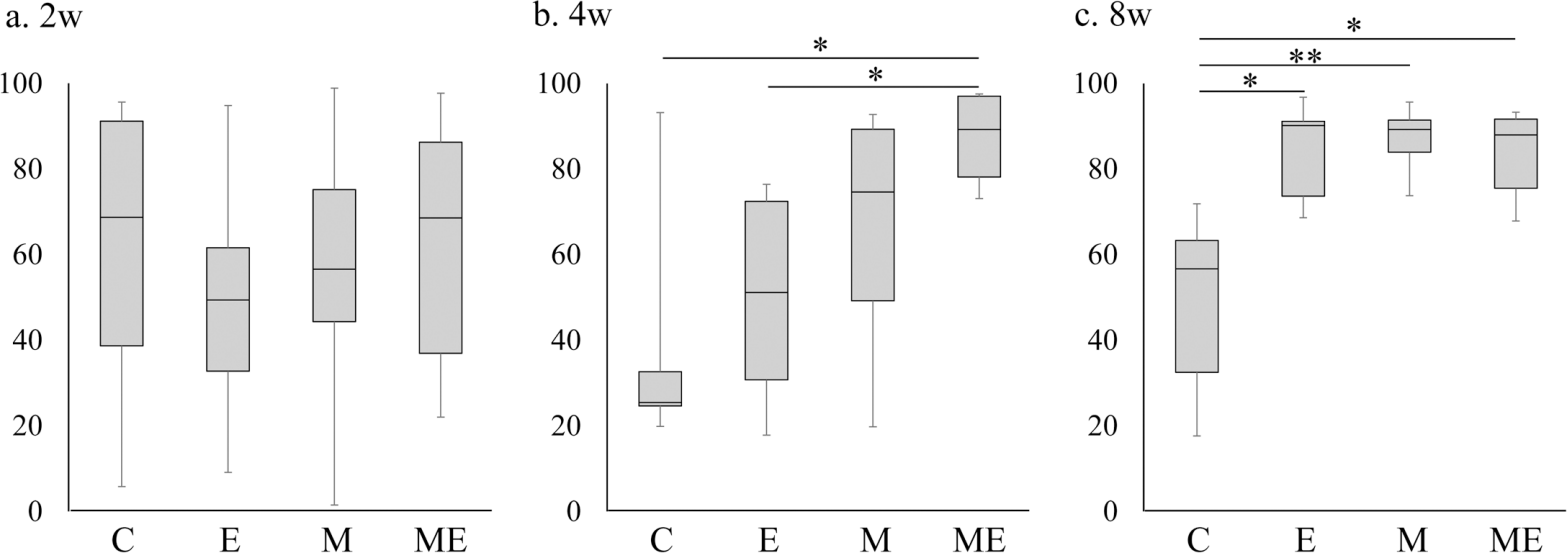
Quantitative evaluation for area% of type II collagen IHC staining. a: At 2 weeks, b: At 4 weeks, c: At 8 weeks. Boxplots display the median and interquartile range, n = 8/group, (*P < 0.05, **; *P* < 0.01).

In the M and ME group the Wakitani cartilage repair score at 2 weeks improved significantly when compared with C group (*P* < 0.05, Fig 5A). At 4 weeks, the score associated with the M group was significantly improved compared with that from the C group (*P* < 0.01, Fig 5B). Furthermore, the scores in the ME group significantly improved compared with the scores in the C (*P* < 0.01), E (*P* < 0.01), and M groups (*P* < 0.05, Fig 5B). At 8 weeks, the scores from the E, M, ME groups all significantly improved compared with C group (*P* < 0.01, Fig 5C). The score of the ME group improved slightly in comparison to the M group, but there were no significant difference at 8 weeks. The breakdown of the Wakitani score parameter is described in Table 2. The quantitative evaluation for % area of type II collagen from the IHC staining, indicated that there were no significant differences at 2 weeks, but that at 4 weeks the ME group % area increased significantly with the C and E groups (*P* < 0.05, Fig 6B). With respect to the % area of type II collagen IHC staining at 8 weeks, the E (*P* < 0.05, Fig 6C), M (*P* < 0.01, Fig 6C) and ME group (*P* < 0.05, Fig 6C) increased significantly compared with the C group.

**Table 2:** Results of 5 parameter and total score in Wakitani score in 2, 4, and 8 weeks. Displacement values were given as median (interquartile). MSC: mesenchymal stromal cell, C: control group, E group: exercise group, M group: MSC injection group, ME group: exercise with MSC injection group. n = 8, (vs. C: *; *P* < 0.05, **; *P* < 0.01, vs. E: †; *P* < 0.05, ‡; *P* < 0.01, vs. M: §; *P* < 0.05).

		C	E	M	ME
2w					
Cell morphology		2 (1–2.25)	2 (1.75–2)	1 (0.75–1.25)	1 (0.75–1.25)
Matrix staining intensity		3 (3–3)	3 (2.75–3)	2 (1.75–3)	2.5 (1.75–3)
Surface regularity		1 (1–2)	1 (1–1.25)	1 (1–1)	0.5 (0–1)
Thickness of cartilage		1 (0.75–2)	1 (1–2)	1 (0.75–1)	1 (0.75–1)
Integration with host cartilage		2 (1–2)	0.5 (0–2)	1 (1–1)	1 (0–1)
Total		9 (8.5–9.25)	7.5 (6.5–9.25)	6 (4.75–7.25)*	5 (4–6)*
4w					
Cell morphology		2 (2–2)	2 (1–2)	1 (0.75–1.25)**	0 (0–0)**
Matrix staining intensity		3 (2–3)	3 (2.75–3)	2 (1.75–3)	1 (1–1.25)
Surface regularity		1 (1–1.25)	1 (0.75–1)	1 (1–1.25)	1 (0.75–1)
Thickness of cartilage		1.5 (0.75–2)	1 (0–2)	0 (0–0)	0.5 (0–1)
Integration with host cartilage		1 (1–2)	1 (0–2)	1 (0.75–1)	1 (0.75–1)
Total		8 (7.75–9)	7.5 (6.75–8)	5.5 (5–6) **, †	4 (3–4.25) **, ‡, §
8w					
Cell morphology		2 (1.75–2)	1 (0–2)	0.5 (0–1)*	0 (0–1)**
Matrix staining intensity		3 (3–3)	2 (2–2)	2.5 (2–3)*	2 (1.75–2.25)**
Surface regularity		1 (1–1.25)	0 (0–1)	1 (0.75–1)	1 (0.75–1)
Thickness of cartilage		1.5 (0–2)	0 (0–1)	0 (0–0.25)	0 (0–0)
Integration with host cartilage		2 (1–2)	1.5 (1–2)	1 (1–2)	1 (0.75–1)
Total		8 (7.75–11)	5.5 (5–6)*	5.5 (4.75–6)*	4.5 (4–5)*

## Discussion

In the present study, we assessed the effect of exercise on early phase of cartilage repair following the intra-articular injection of MSCs into rat knees with an osteochondral defect. It was confirmed that defective hyaline cartilage could not be repaired spontaneously (Figs 1E–1H, 2A, 3A and 4A) and the improvement of cartilage repair scores occurred by 2 weeks after the injection of MSCs (Fig 5A). A previous study reported that significant cartilage repair score improvement occurred after the MSC injection in the presence of osteochondral defects and gradually improved after 4–12 weeks. [23] The current study used on the initial change between the MSC injection time point and 8 weeks post-injection, and it was noted that exercise may not inhibit the effect of articular cartilage repair score improvement induced by the intra-articular injection of MSCs. This was evident as improvement in the cartilage repair scores was only slightly promoted by exercise (Fig 5). The initiation of exercise 2 days after the MSC injection might facilitate cartilage repair score improvement and may improve type II collagen synthesis while prohibiting the production of type I collagen; a finding that was not evident in the groups that did not receive an injection or those that received an injection but did not exercise. In this study, no significant difference in the scores was observed among the E, M, and ME groups at 8 weeks. In the previous study, exercise performed 4 weeks after the osteochondral defect was created promoted healing of the cartilage defect in 6 and 10 weeks after creation of the defect.[22] In this study, exercise performed 4 weeks after the defect was created improved the cartilage repair score at 8 weeks after initiation of the exercise intervention. On the other hand, the repaired tissue in the ME group showed slightly reduced SO staining intensity and a low total score. The tissue repaired by MSC injection and exercise intervention might be at a risk for repaired cartilage degeneration in the future.

It has been reported that exercising on a treadmill at a moderate speed suppresses cartilage ECM degeneration [15] and chondrocyte apoptosis.[16] In these reports, a moderate level of exercise consisting of running on a treadmill set at 12–18 m/min for 30 min was considered the chondroprotective level for rat knees; however, there is little evidence regarding the effect of exercise following cell supplementation therapy *in vivo*. In a study of cartilage repair, continuous passive motion influenced the induction of neochondrogenesis in free intra-articular periosteal autografts compared with the findings in immobilized grafts.[26] Previous studies have also reported that MSCs are associated with mechanical stress *in vitro*, which may explain the growth-enhancing effect of exercise.[19] MSCs stimulate cell proliferation and chondrogenic differentiation [27] and lead to anti-inflammatory activities, such as decreased gene expression of interleukin-1 beta and matrix metalloproteinases 1 and 13.[28] One *in vitro* study reported that cyclic compressive loading could promote chondrogenesis in bone marrow-derived MSCs by inducing the synthesis of TGF-β1, which can stimulate MSCs to differentiate into chondrocytes.[29] The chondrogenic differentiation of MSCs requires a chemical or mechanical stimulus.[30] During articular cartilage development, physiological loading on the joint is an important factor influencing the differentiation of MSCs into chondrocytes and a mechanical stimulus on chondrocytes is essential for the maintenance of cartilage integrity.[29, 31, 32] In addition, cartilage has been reported to be affected *in vivo* by mechanical stresses, such as shear stress or load bearing.[33] In the current study, while 4 weeks of prolonged moderate exercise promoted type II collagen synthesis (Figs 3B–3E, 3D and 3E and 6B), the absence of exercise, limited the synthesis of type II collagen in an MSC injected osteochondral defect model. This result indicates that mechanical stress induced by a moderate level treadmill exercise might affect the cartilage repair process. Song et al. suggested that exercise 2 weeks after osteochondral injury would further deteriorate the cartilage defects, whereas exercise 4 weeks after osteochondral injury could promote cartilage ECM synthesis.[22] MSC is a highly adherent cell that requires several hours to days to adhere to the tissue.[34] To avoid exercise-mediated disruption of cell adherence to the host tissue, we did not initiate exercise until 2 days after the intra-articular injection. This study confirmed that initiation of exercise 2 days after the MSC injection promotes cartilage repair, but an earlier or later initiation of exercise would exert different effects as those observed in the current study. Further studies will be needed to investigate other protocols for exercise or mechanical stimulation. Therefore, it is necessary to explore the appropriate time to initiate exercise. Song et al. indicated that exercise from 4 to 10 weeks following surgery to correct an osteochondral defect could stimulate endogenous MSCs to regenerate defective cartilage.[22] In the current study, the cartilage repair score associated with the exercise without MSC injection group at 8 weeks (that performed exercise 4–12 weeks after osteochondral defect) improved compared with that in the control group (no exercise group). There was no significant differences between the cartilage repair score of the exercise without MSC injection group and the MSC injection group. However, type I collagen expression in the repaired tissue, which represents fibrocartilage tend to decrease in the ME group (Fig 4B–4F and 4D–4F). This may indicate that MSC injection yields good ECM production for cartilage repair and when combined with exercise might assists in tissue repair; however, it is unclear whether the beneficial effect is time-dependent.

Interestingly, the immature bone in the C group was found to contain type II collagen at each weeks in this study. Thus, the pace of subchondral bone reconstitution differed according to the intervention and MSC injection, and exercise intervention might promote subchondral bone reconstitution. Both the cartilage and subchondral bone play an important role in shock absorption and support the joints. MSC can differentiate into chondrocytes and osteocytes. Their differentiation fates are determined by circumstances, and mechanical stress is one of the determining factor.[35] Cartilage repair and subchondral bone reconstitution occur at different paces, and advancement of the subchondral bone does not diminish articular cartilage repair, as observed in an osteochondral defect model.[36] Future studies should evaluate the parameters of subchondral bone reconstitution and cartilage repair.

This study had several limitations. First, the rats employed in this study were 8 weeks old which represents an age of skeletal immaturity. It is generally accepted that immature animals have an increased capacity to heal compared with mature animals.[37] However, in the current study, the result of the C group at 2, 4 and 8 weeks suggested that the animals had a limited capacity for spontaneous recovery in defective cartilage while the results of the M and ME groups were suggestive of improvements in cartilage repair. Second, we did not clarify whether repaired cartilage was derived from injected MSCs because they were not labeled with a cell tracer. In the model used here, surgery to create osteochondral defects promotes the movement of endogenous MSCs into the intra-articular joint, which might stimulate cartilage repair. However, a previous study [23] reported injected MSCs that were traced until after 4 weeks and the results of the M and ME groups compared with those of the C and E groups indicated that cartilage repair was promoted by both native progenitor cells contained in cartilage [38] and injected MSCs in addition to some kinds of cytokine which MSCs release for cartilage formation. Third, osteochondral defects were created in both the right and left femoral groove, which is an indirect weight-bearing area. Therefore, it is essential in a future study to determine the appropriate method of mechanical stimulation for direct loading areas. In this study protocol, osteochondral defects were created in both the knees of the rats to reduce the number of rats used, in accordance to the animal ethics regulations. The rats could load the weight on their preferred leg and thereby load lesser weight on the other leg. The effect of activity on both knees might be interacted each other. If either the right or left hind limb were operated on, the results would differ from the results of the current study.

## Conclusions

In conclusion, exercise performed after the intra-articular injection of MSCs for osteochondral defects could efficiently lead to cartilage repair in a rodent osteochondral defect model. Further experiments are necessary to elucidate the mechanisms that exercise stimulates in the MSCs in order to different into chondrocytes or to omit some kind of secretion which may contribute to cartilage repair.

## Supporting Information

S1 Fig Chondrogenic potential of the bone marrow mesenchymal stromal cells (MSCs) was examined by a pellet cultures. Approximately 2.5 × 105 cells, passage 3, were cultured in differentiation basal medium-chondrogenic (Lonza, Maryland, USA) with transforming growth factor-beta3 (R&D Systems, Minesota, USA) and centrifuged in a 15-ml polypropylene tube to form a pellet. The pellet was observed in macroscopic and histologic after 3 weeks cultured in pellet. a: macro image of pellet-cultured MSCs. White bar represents 1 mm. b: histological image stained with 1.5% Safranin-O/fast green. c: immunohistochemical staining of type II collagen by DAB. White bar and black bars each represent 0.5 mm.
(TIF)

S2 Fig All histological and IHC staining images of C group at 2 weeks. (TIF)

S3 Fig All histological and IHC staining images of E group at 2 weeks. (TIF)

S4 Fig All histological and IHC staining images of M group at 2 weeks. (TIF)

S5 Fig All histological and IHC staining images of ME group at 2 weeks. (TIF)

S6 Fig All histological and IHC staining images of C group at 4 weeks. (TIF)

S7 Fig All histological and IHC staining images of E group at 4 weeks. (TIF)

S8 Fig All histological and IHC staining images of M group at 4 weeks. (TIF)

S9 Fig All histological and IHC staining images of ME group at 4 weeks. (TIF)

S10 Fig All histological and IHC staining images of C group at 8 weeks. (TIF)

S11 Fig All histological and IHC staining images of E group at 8 weeks. (TIF)

S12 Fig All histological and IHC staining images of M group at 8 weeks. (TIF)

S13 Fig All histological and IHC staining images of ME group at 8 weeks. (TIF)

S1 Table All histological scoring data which were evaluated by the Wakitani cartilage repair score. (XLSX)
