# Supplementary figures and images for: The Effect of Exercise on the Early Stages of Mesenchymal Stromal Cell-Induced Cartilage Repair in a Rat Osteochondral Defect Model
Source: PLoS One. 2016 Mar 11;11(3):e0151580. doi: 10.1371/journal.pone.0151580 (PMC4788414; doi:10.1371/journal.pone.0151580)

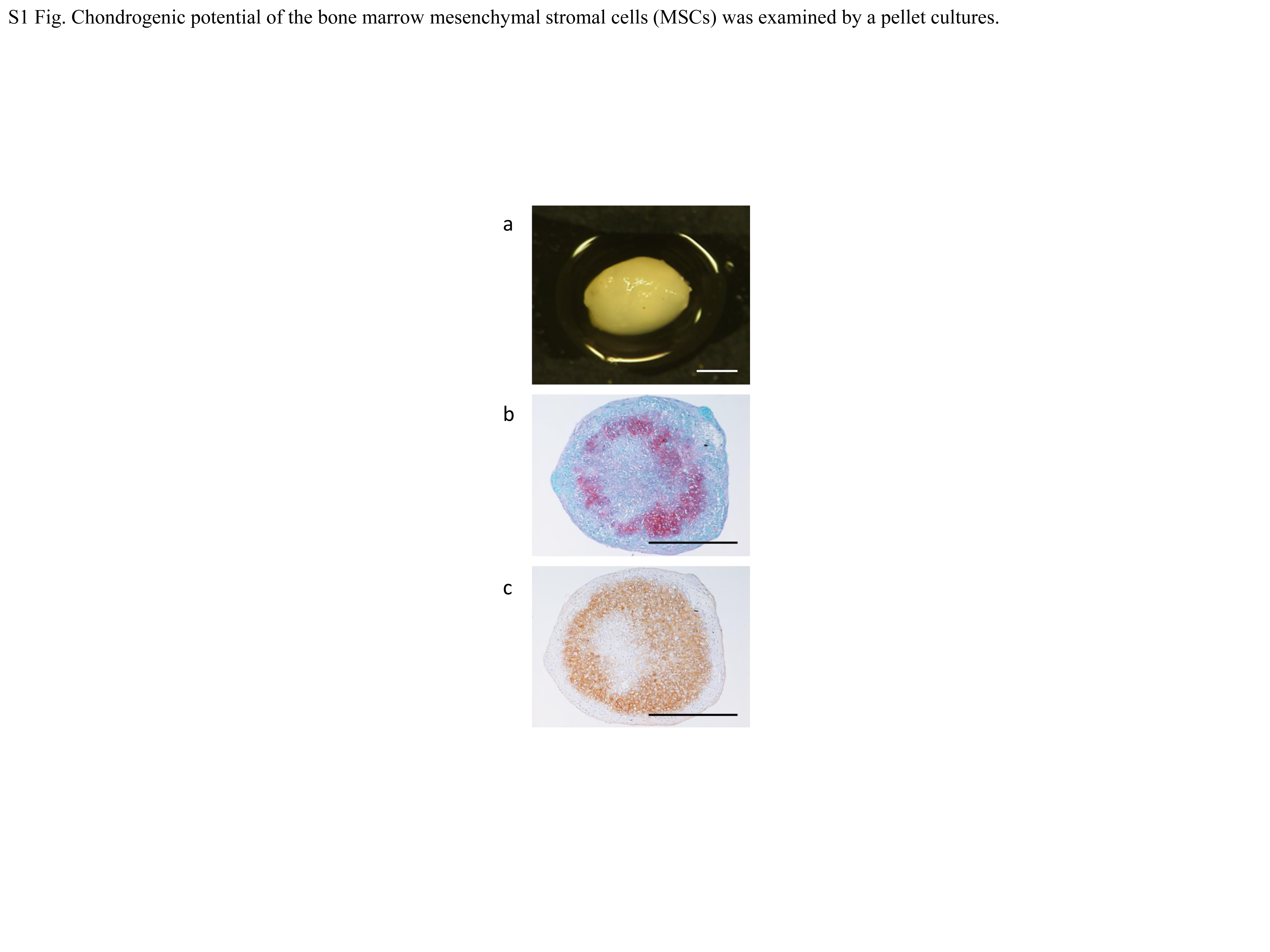

Supplement: S1 Fig — Approximately 2.5 × 10^5^ cells, passage 3, were cultured in differentiation basal medium-chondrogenic (Lonza, Maryland, USA) with transforming growth factor-beta3 (R&D Systems, Minesota, USA) and centrifuged in a 15-ml polypropylene tube to form a pellet. The pellet was observed in macroscopic and histologic after 3 weeks cultured in pellet. a: macro image of pellet-cultured MSCs. White bar represents 1 mm. b: histological image stained with 1.5% Safranin-O/fast green. c: immunohistochemical staining of type II collagen by DAB. White bar and black bars each represent 0.5 mm. (TIF) [file pone.0151580.s001.tif]

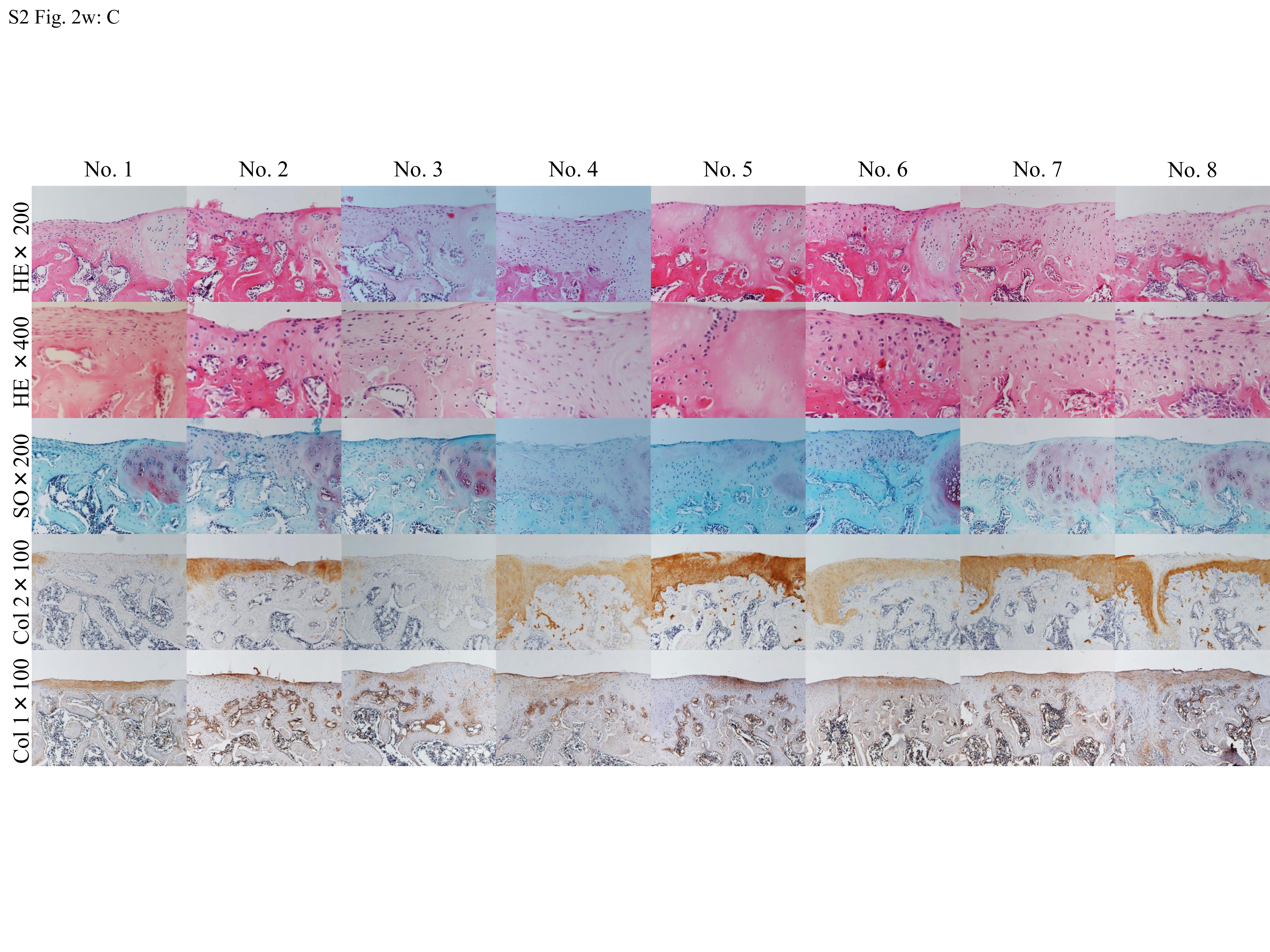

Supplement: S2 Fig — (TIF) [file pone.0151580.s002.tif]

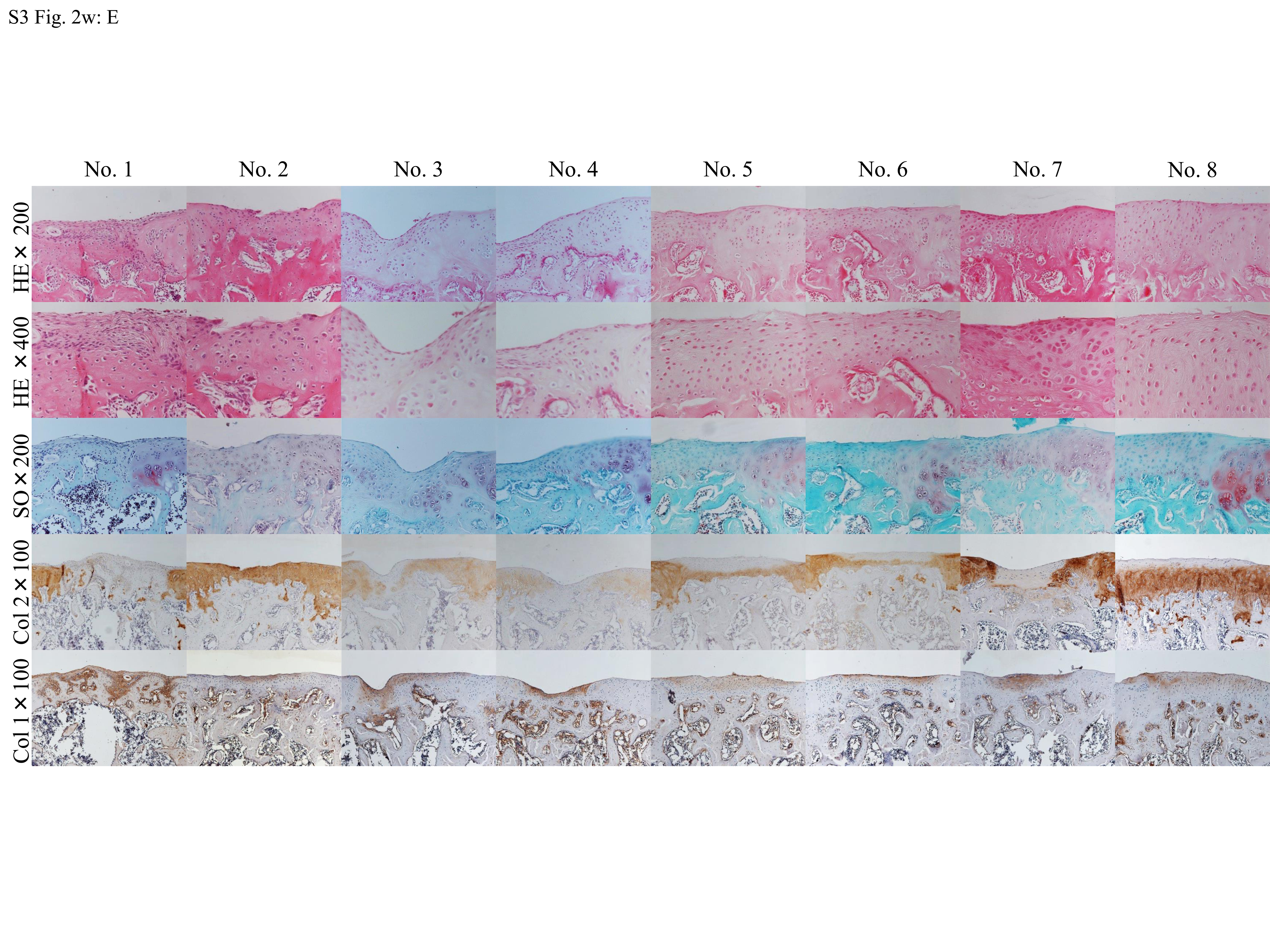

Supplement: S3 Fig — (TIF) [file pone.0151580.s003.tif]

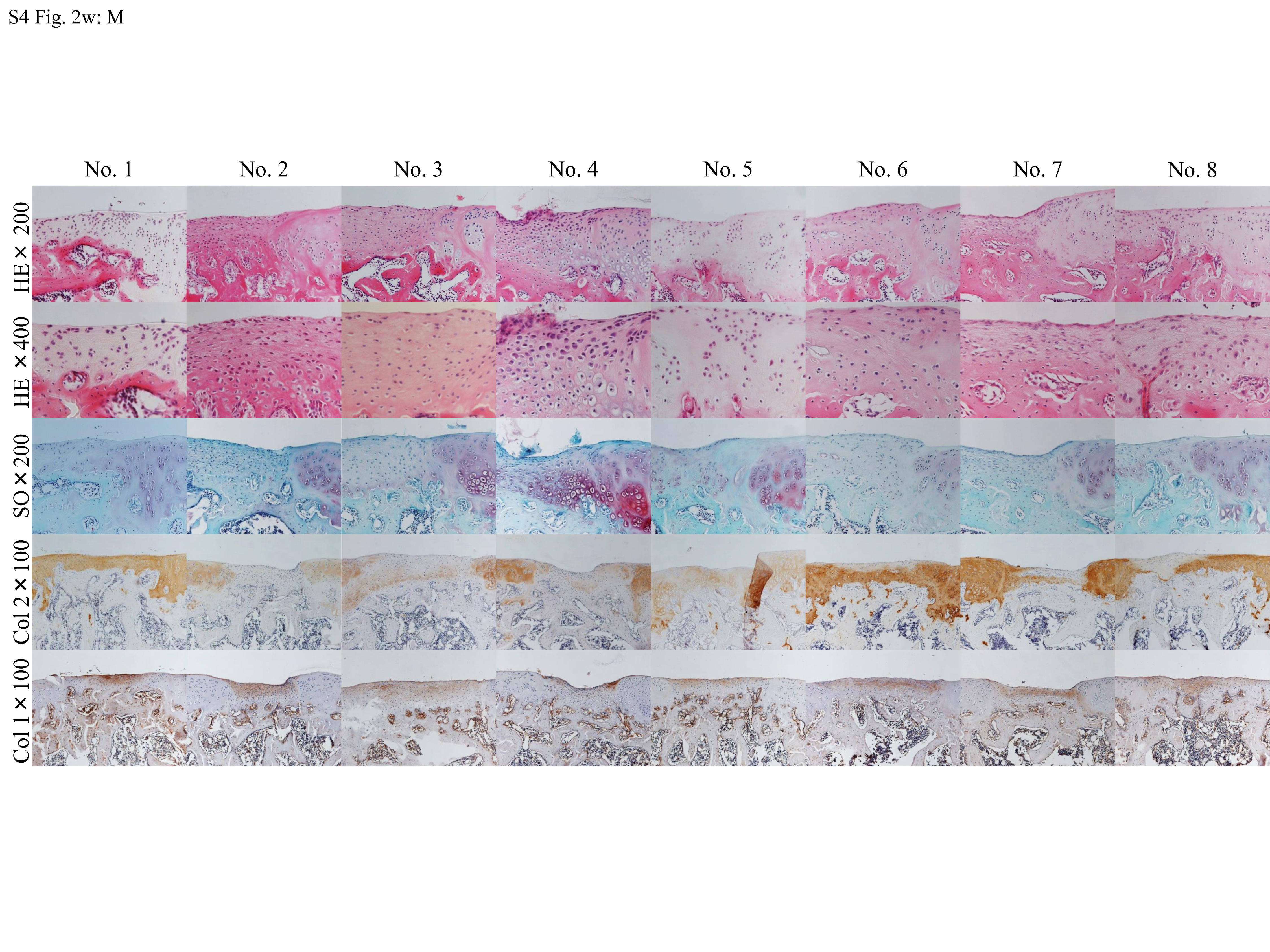

Supplement: S4 Fig — (TIF) [file pone.0151580.s004.tif]

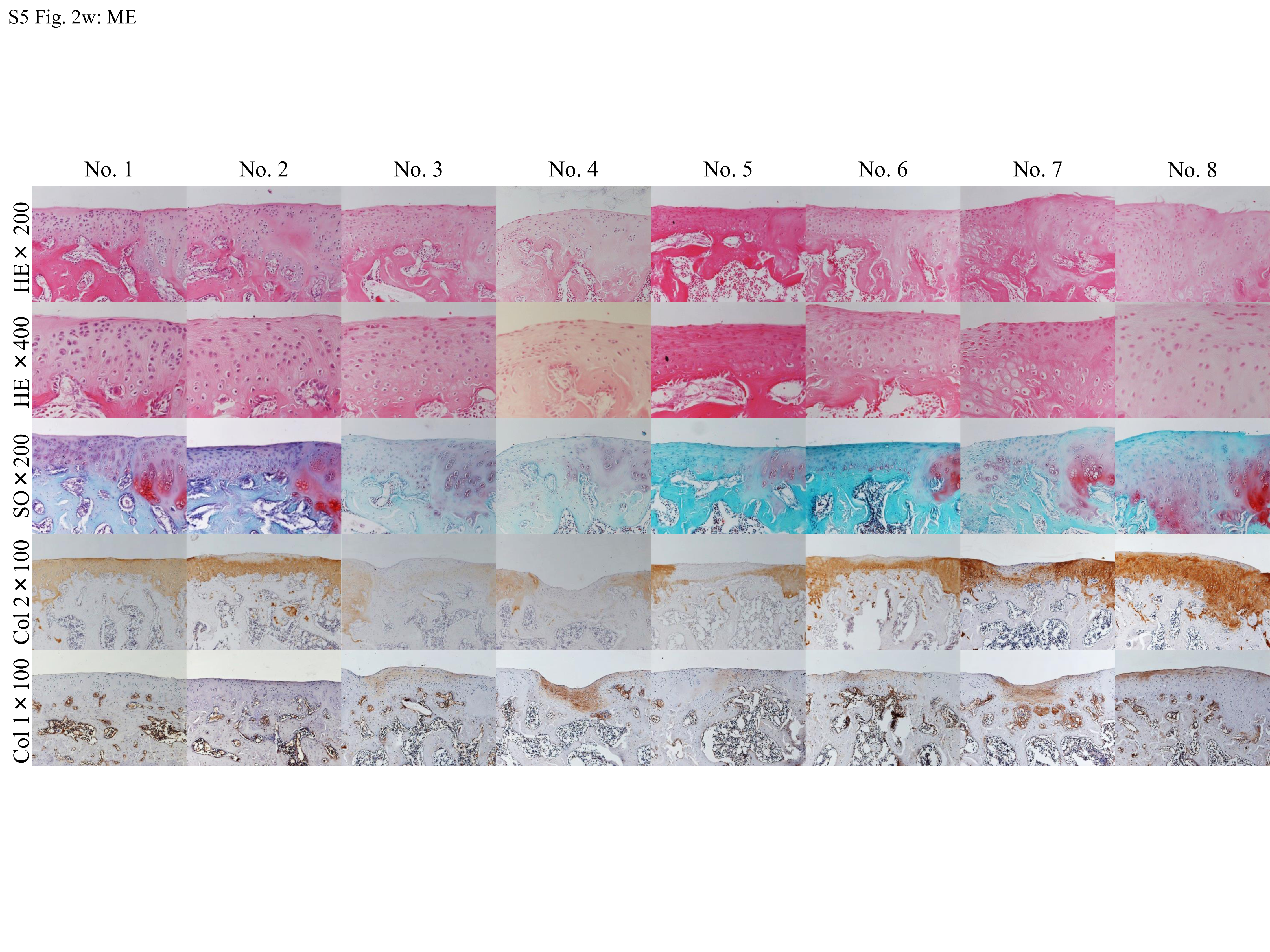

Supplement: S5 Fig — (TIF) [file pone.0151580.s005.tif]

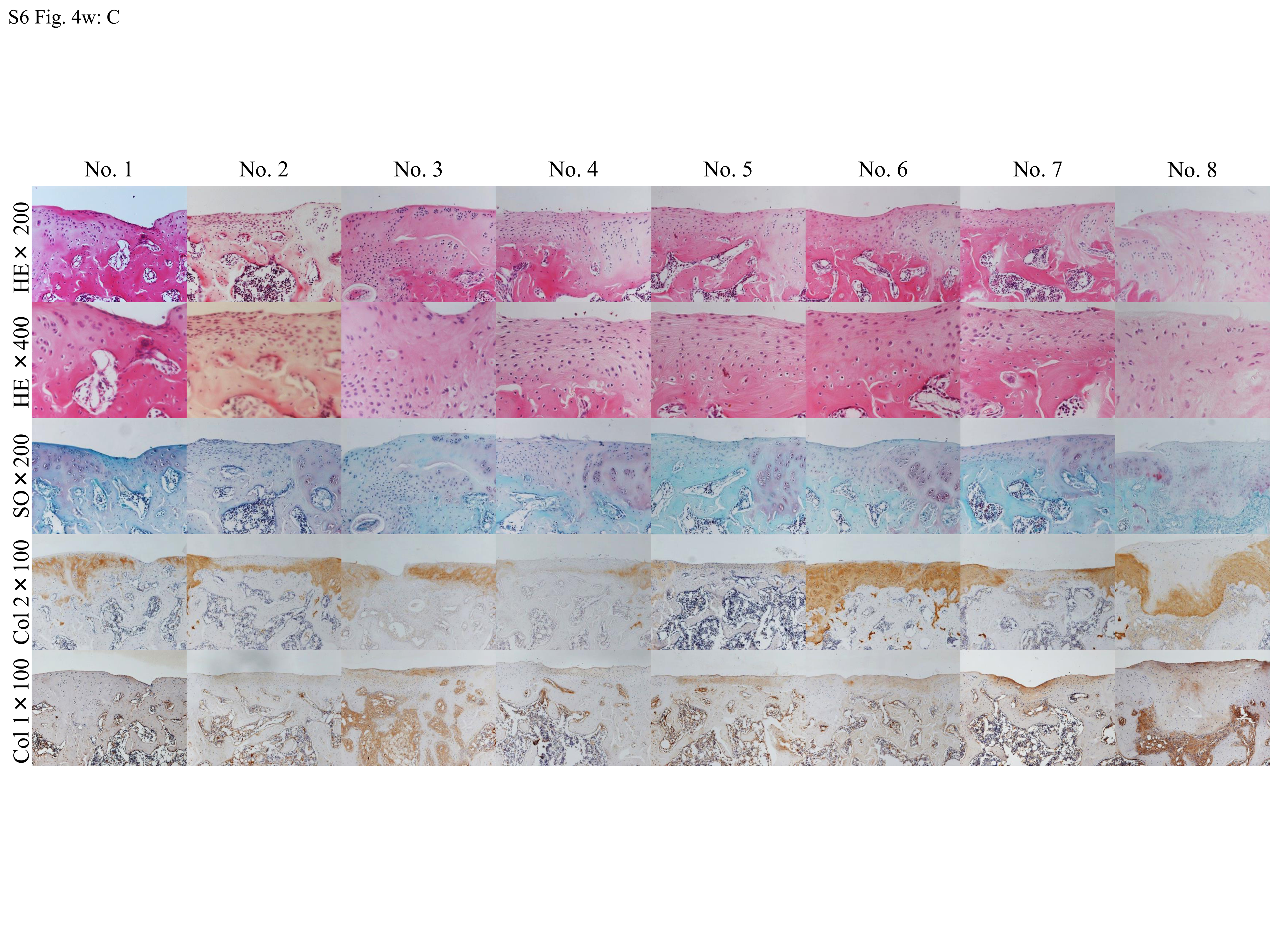

Supplement: S6 Fig — (TIF) [file pone.0151580.s006.tif]

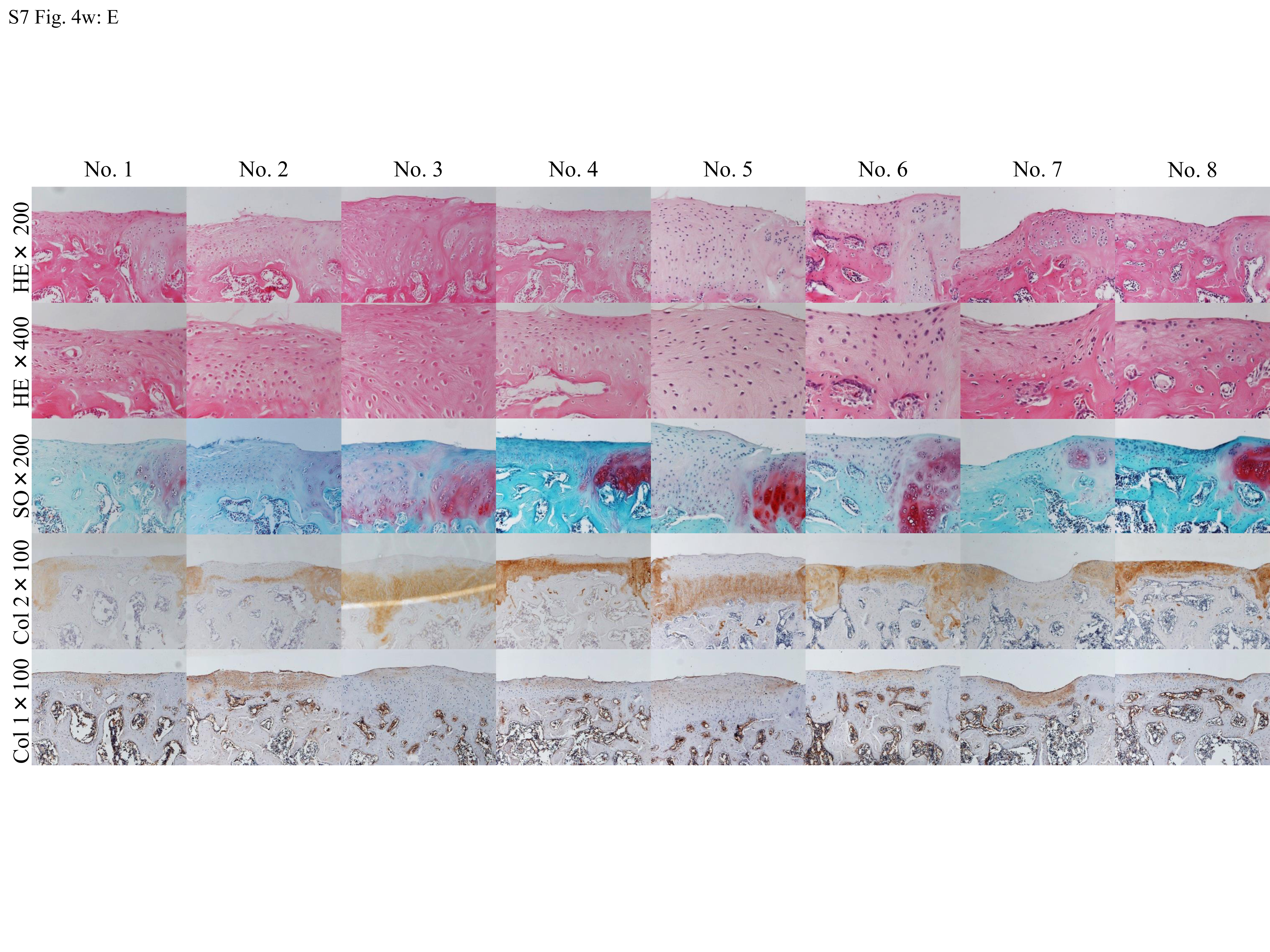

Supplement: S7 Fig — (TIF) [file pone.0151580.s007.tif]

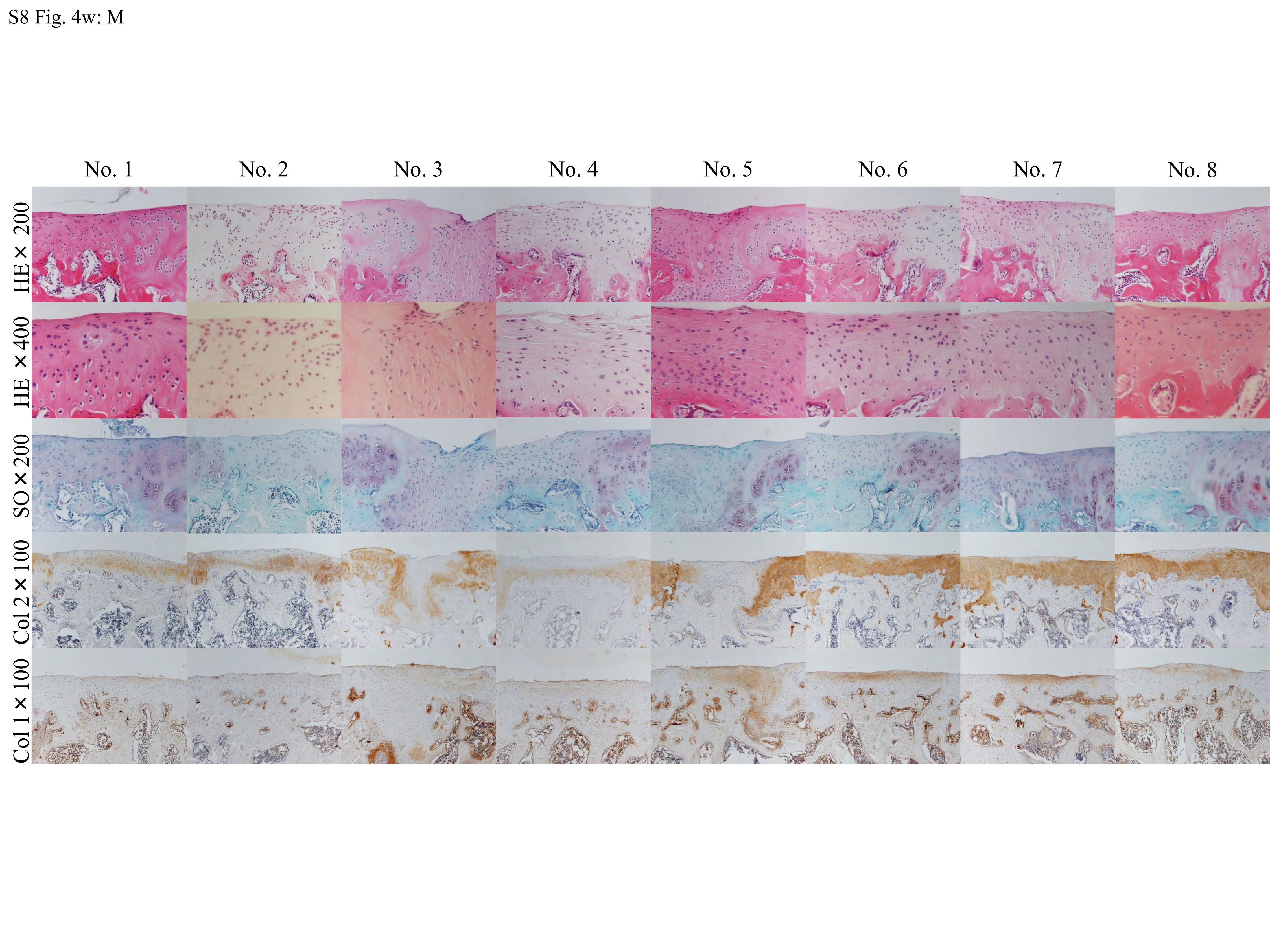

Supplement: S8 Fig — (TIF) [file pone.0151580.s008.tif]

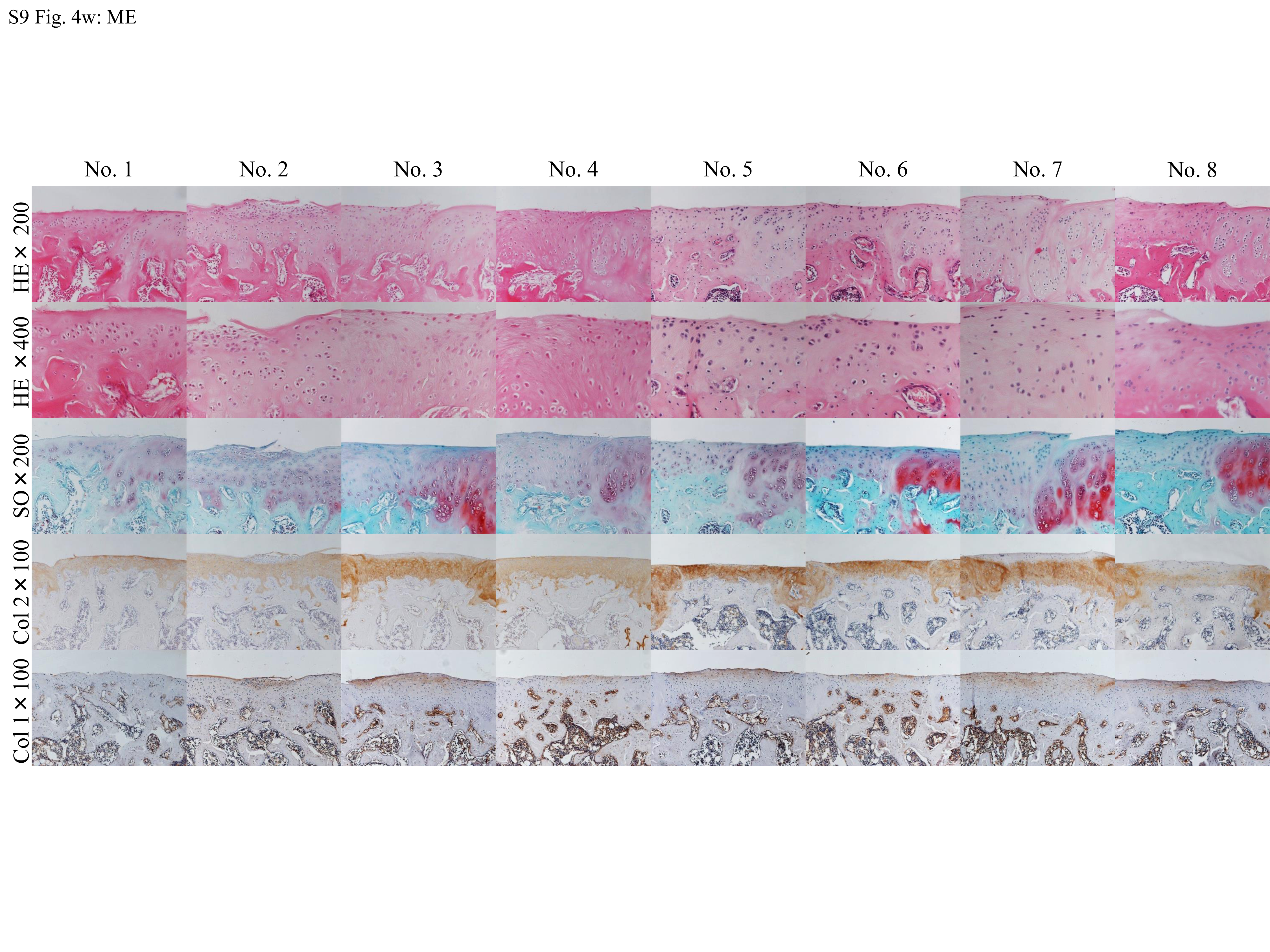

Supplement: S9 Fig — (TIF) [file pone.0151580.s009.tif]

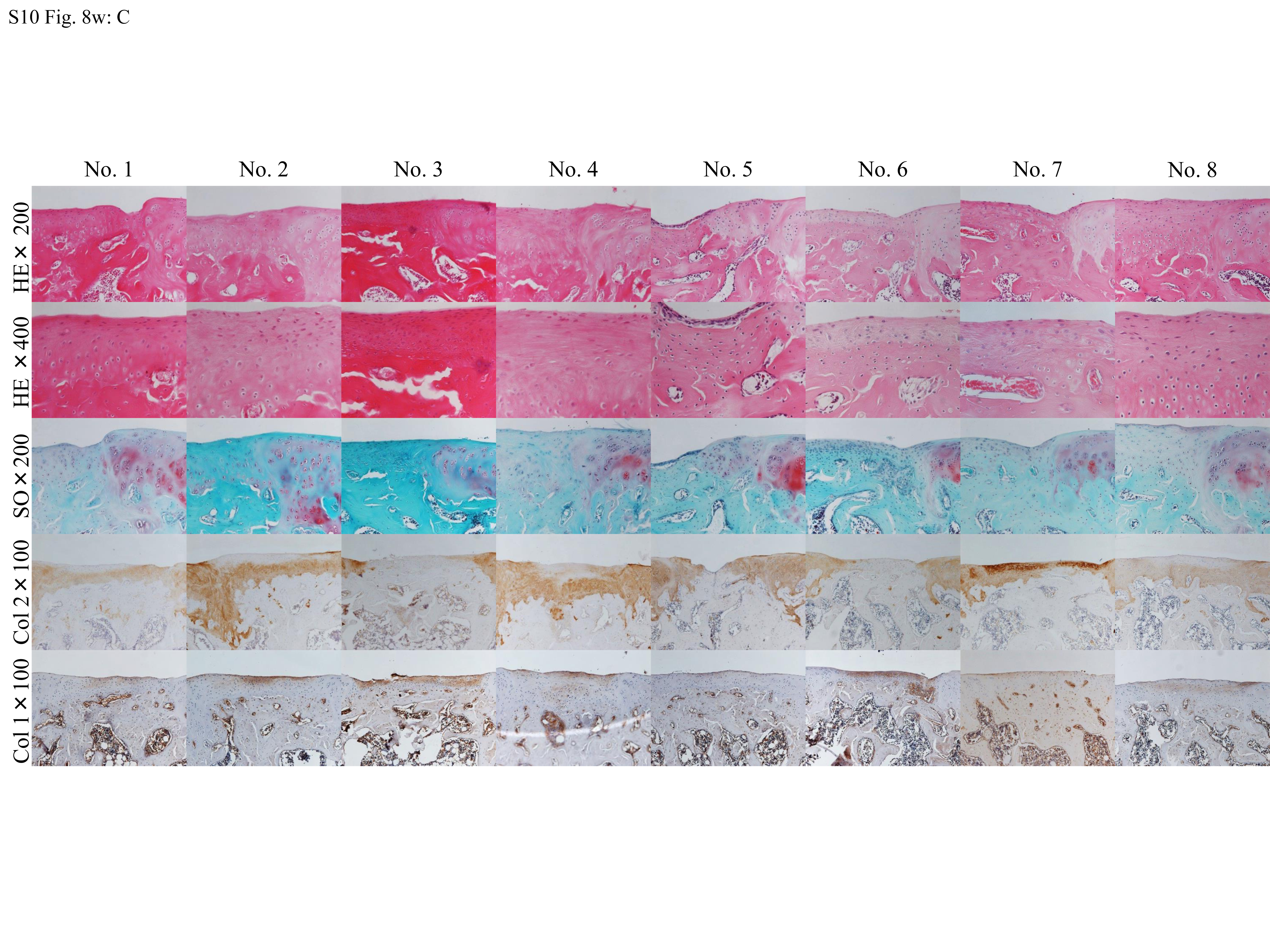

Supplement: S10 Fig — (TIF) [file pone.0151580.s010.tif]

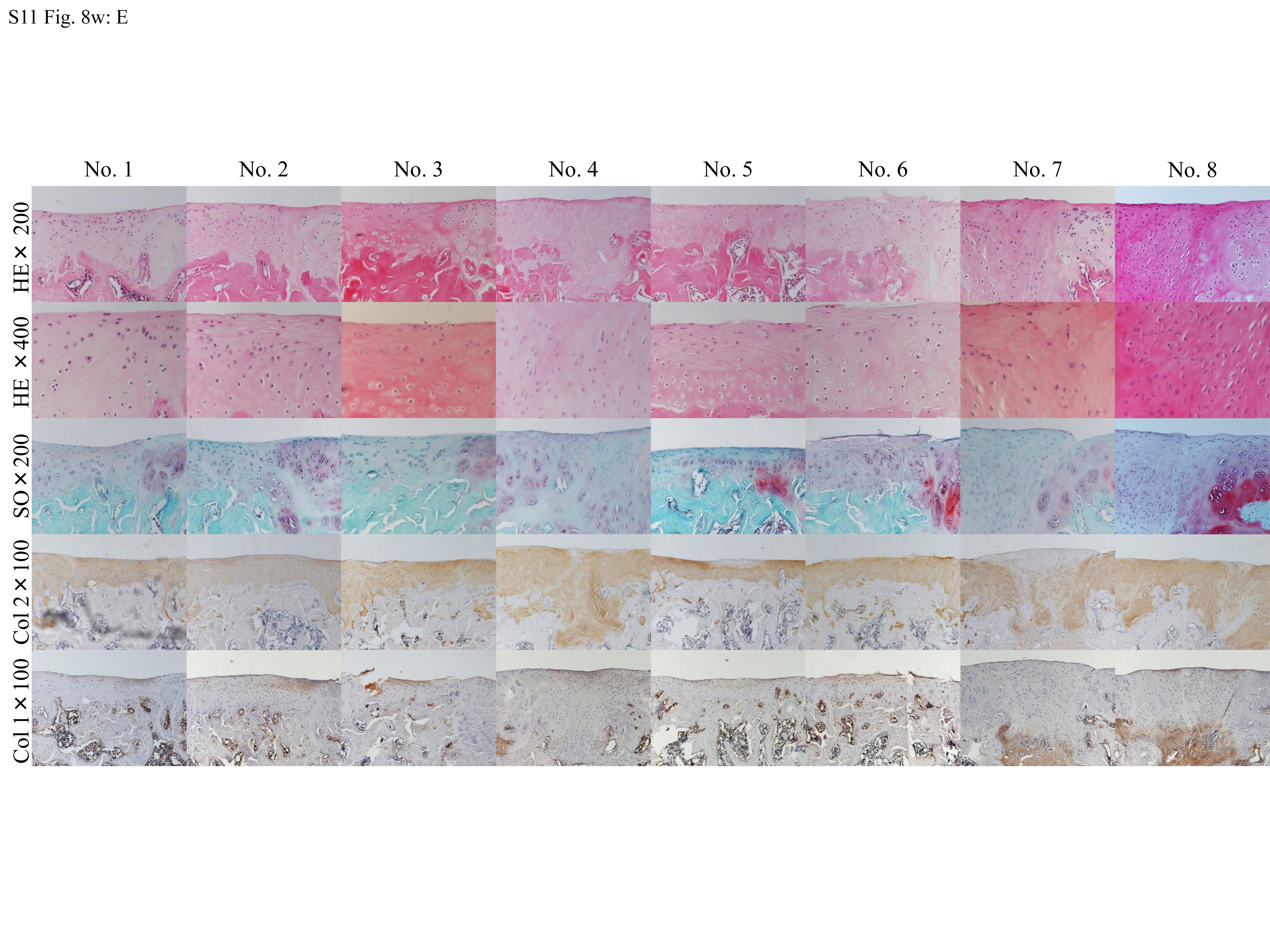

Supplement: S11 Fig — (TIF) [file pone.0151580.s011.tif]

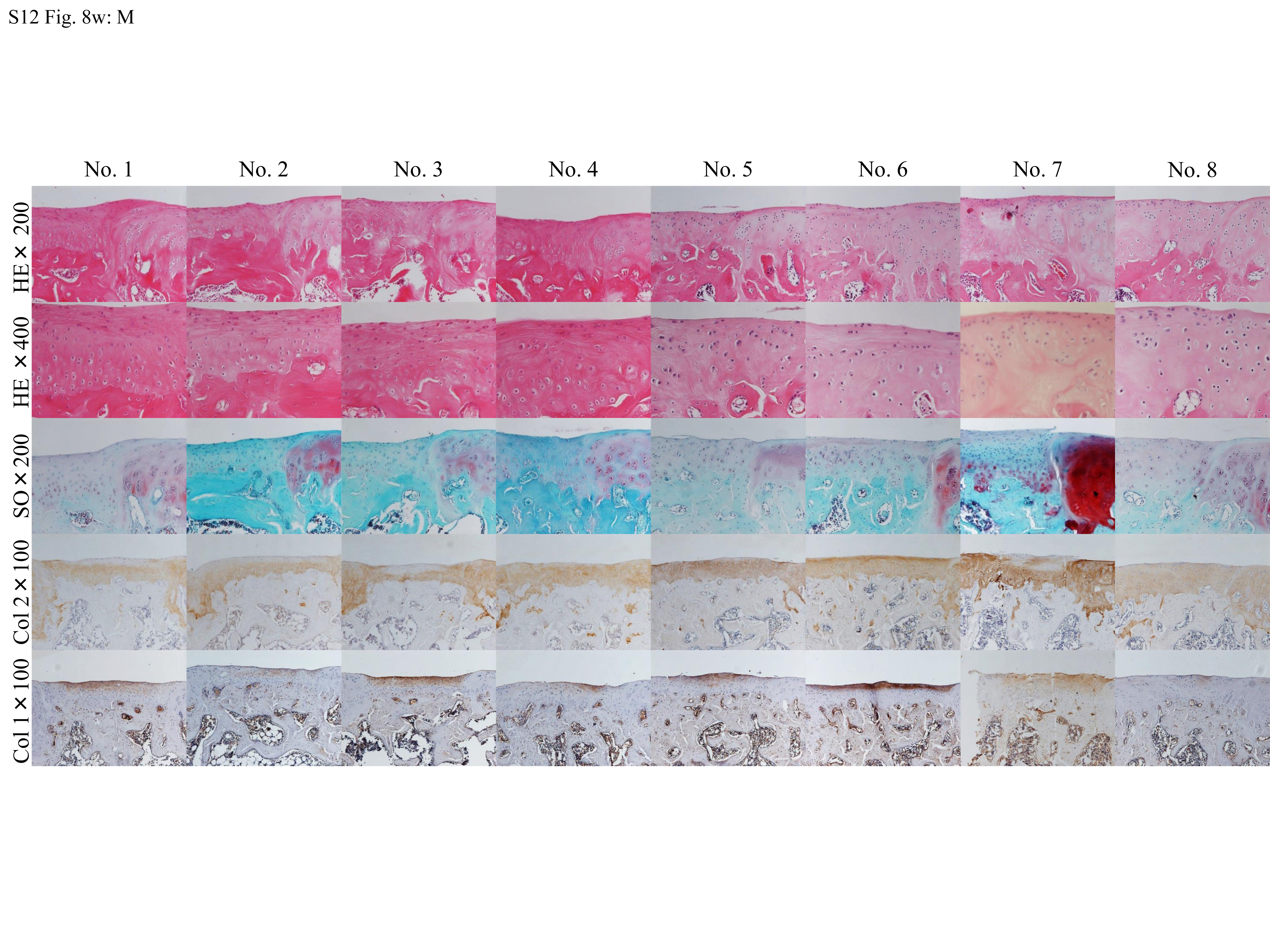

Supplement: S12 Fig — (TIF) [file pone.0151580.s012.tif]

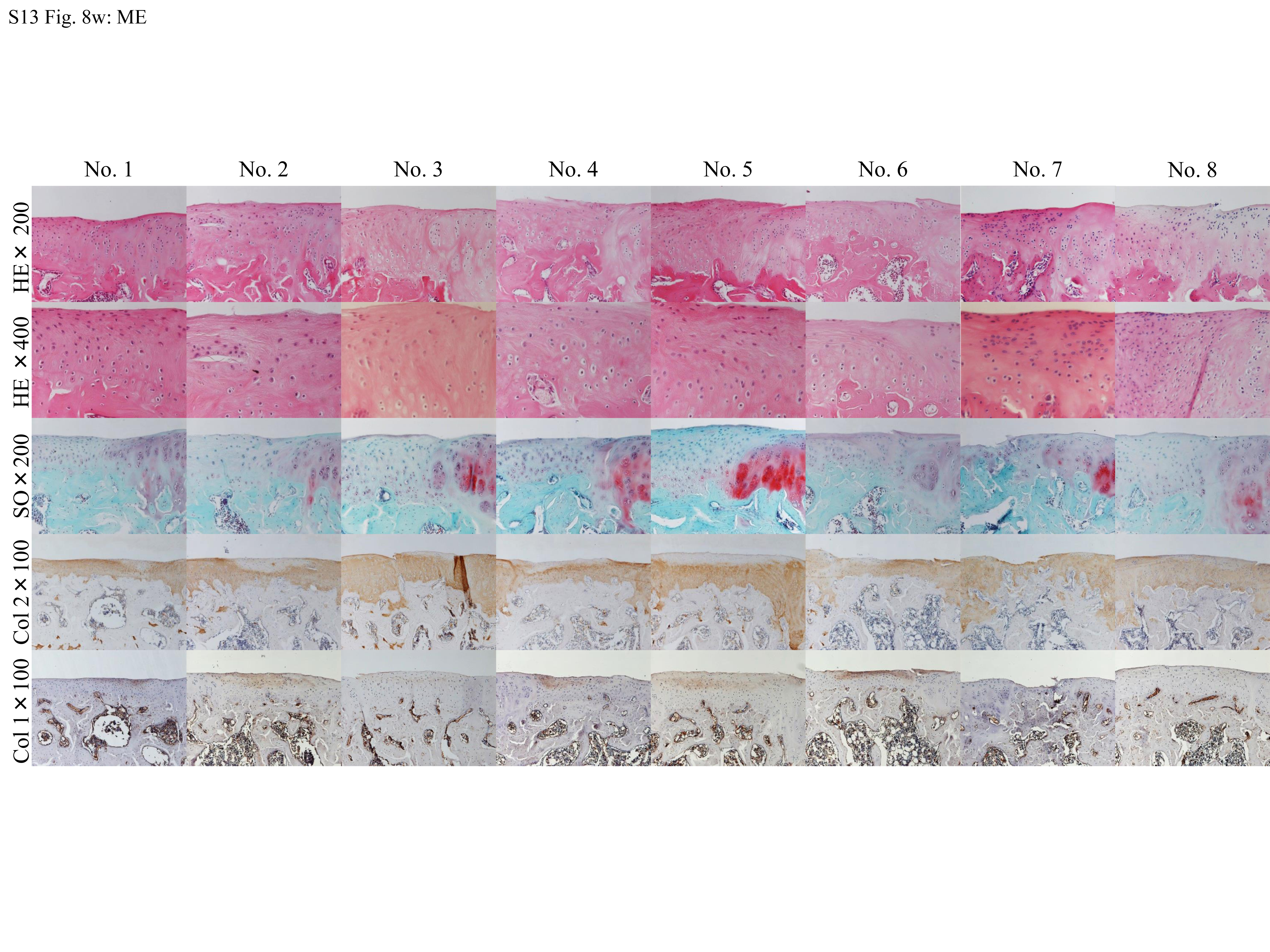

Supplement: S13 Fig — (TIF) [file pone.0151580.s013.tif]
